# Social Norms: A Missing Ingredient of Programs Seeking to Foster Women’s Agency in Nutrition

**DOI:** 10.1016/j.cdnut.2024.104440

**Published:** 2024-08-14

**Authors:** Francine E Wood, Katherine L Dickin, Lisa Sherburne, Mariam Diakite, Abdoulkader Boubacar, Meghan Pollak, Rebecka Lundgren

**Affiliations:** 1 Center on Gender Equity and Health, University of California San Diego, La Jolla, CA, United States; 2 Department of Public & Ecosystem Health, Cornell University, Ithaca, NY, United States; 3 USAID Advancing Nutrition/The Manoff Group, Arlington, VA, United States; 4 Center on Gender Equity and Health, University of California San Diego, Bamako, Mali; 5 Save the Children, Maradi, Niger; 6 Save the Children, Fairfield, CT, United States

**Keywords:** agency, social norms, social context, community engagement, social and behavior change, nutrition programming and research, maternal nutrition, infant and child nutrition

## Abstract

Social expectations play a crucial role in shaping dietary practices among women and children. However, despite significant attention to promoting social and behavioral change in nutrition-focused programs and research, the influence of social norms on women’s agency in enhancing nutrition practices is often overlooked. In this perspective, we advocate for a paradigm shift by incorporating a “norms aware” approach. This underscores the importance of recognizing, measuring, and addressing the societal constraints and barriers that women and children encounter in their journey to improved nutrition. Drawing on insights from the United States Agency for International Development-funded Kulawa project in Niger, we highlight the implications of using social norms diagnosis tools to understand the contextual dynamics within child-feeding practices, informing intervention design, and targeted populations. Integrating a norms perspective into nutrition programming and research does not require an overhaul, but rather a nuanced application of understanding of contextual drivers, such as social norms and agency, that have been underemphasized. We delve into the role of the socio-ecologic system, underscore the importance of addressing power imbalances related to gender and social hierarchy, and emphasize that programs targeting norms should aim for community rather than individual-level change. We provide guidance for programs and research integrating a norms perspective, as well as examples of how tools, such as the Social Norms Exploration Tool and Social Norms Analysis Plot framework, can be applied to identify and prioritize social norms, facilitating the design of “norms aware” programs. Additionally, we highlight the critical role of community engagement and discuss the value of using qualitative and quantitative approaches to document the process and outcomes of social norms research, program design, and implementation. When we recognize the role of social norms in nutrition as a missing ingredient in nutrition research, programming, and social and behavior change strategies, we create opportunities for more effective and contextually relevant research and interventions that address the complexities of enhancing nutrition practices among women and children.

## Norms: A Missing Ingredient in Nutrition Programs?

Despite decades of nutrition programming intended to address undernutrition, 22.3% of children are stunted and 6.8% of children are wasted worldwide [1], limiting health and development around the globe. Children in low- and middle-income countries are disproportionately affected, with 4.7% of children experiencing both stunting and wasting; in parts of South Asia and sub-Saharan Africa, even more children are undernourished [1,2]. Poverty and food insecurity underlie much of the world’s undernutrition, yet solutions include not only access to material resources, food, and health services but also a supportive social environment. Although there is evidence of the efficacy of improving infant and young child-feeding practices, maternal micronutrient supplementation, and food fortification [3,4], less is known about how to design and deliver effective programs that enable the uptake and maintenance of recommended nutrition behaviors [5,6]. In addition to the need for high-quality “how to” research built into the design of nutrition programs and adaptive learning to better understand what does not work [7], greater attention to enabling environments and behavioral drivers is needed.

The impact of social and behavioral change interventions often focused on informing and motivating women to adopt new dietary practices for themselves and their children, can be limited by a lack of agency – the ability to set and act on self-determined goals. Although programs call on women to take action, the social and gender norms upheld by families, communities, and leaders not only directly impact food choice and allocation, but also critically impact women’s agency to improve nutrition practices [5,8,9]. Social norms are the perceived informal rules, unwritten and unspoken, that govern what behaviors are typical, acceptable, and appropriate within a given group [10,11]. The influence of social norms in creating enabling environments for feeding and care is recognized in conceptual frameworks on maternal and child nutrition [12,13], along with governance and resources. Yet social and behavior change research and programming for nutrition often overlook social norms, in part because of the challenges of identifying and measuring relevant norms, or the tendency to prioritize individual attitudes and skills, or to focus solely on structural-level factors without understanding community rules and shared expectations and beliefs. The purpose of this perspective was to call for more attention to social norms within nutrition research and interventions, as a means to increase the effectiveness of efforts to support women’s agency and thereby improve nutrition behaviors to prevent undernutrition and support family well-being.

## Norms Shape Agency in Multiple Ways

Social norms exist within a web of culture, can be positive or negative, and often shift over time. Norms that operate across all elements of human experience (e.g., family and community relationships, livelihoods, religion, health, etc.) and influence multiple behaviors, often referred to as meta-norms, exist and interact across multiple levels of the socio-ecologic system [14,15]. These meta-norms are deeply rooted and may be more resistant to change than proximal norms [14]. In the context of nutrition, for example, meta-norms related to gender and authority can influence women’s expectations to prioritize their partner and children’s needs during food distribution within the household [14,16].

Social norms are pivotal in shaping an individual’s agency, which stands at the center of the empowerment process. The agency is the ability of an individual or group to cultivate consciousness of choice, foster self-efficacy, and take agentive action, allowing the pursuit of self-determined goals within the opportunities and constraints of social and institutional structures [17]. The conceptualization of agency and empowerment is nonlinear and multifaceted and encompasses internal attributes such as intra-group dynamics and psychology of the individual or collective, as well as external factors such as social structures and institutions, intergroup dynamics, and community and household dynamics. These factors, together with social norms, can either facilitate or impede agency and empowerment and ultimately, an individual’s ability to perform a behavior such as complementary feeding and breastfeeding [18].

## Nutrition Experiences Responding to Norms and Agency

A review of social norms in the context of complementary feeding in low- and middle-income countries [5] found references across cultures and contexts of norms, beliefs, and expected behaviors related to what children are fed, as well as when, how, and by whom. Only a subset of these papers described intervention approaches intended to address these norms. In a more recent review, Litvin et al. [19] found little attention to social norms in the design, implementation, and evaluation of interventions to improve women’s dietary intake and prevent undernutrition. Although there were some reports of food-specific norms (e.g., taboos or special foods provided during pregnancy or postpartum), the social norms most relevant to women’s nutrition were norms related to gender and family roles, and hence to women’s agency. In both reviews, intervention strategies, when reported, included approaches such as engaging husbands and mothers-in-law during home visits or counseling; gender-transformative dialog in support groups, media, or community events; and mobilizing influential community change agents [5,19].

Although the reviews found growing attention to social norms in more recent publications, few papers described actual approaches to shift or build on norms – or measures of the effectiveness of these approaches. This could reflect a lack of awareness of the importance of social norms in shaping nutrition-relevant behaviors, but even when awareness exists, the development and implementation of intervention approaches can be hindered by a lack of tested strategies to address norms, hesitation to target beliefs or attitudes seen as rooted in local culture, and skepticism about the ability to shift norms in the relatively short time frame of most interventions.

On the other hand, qualitative and participatory research often uncovers ways in which the normative beliefs of family and community members – and women’s own internalized ideals – present barriers or facilitators to the uptake of key nutrition recommendations [20,21]. For example, in many contexts, there is variation in decision-making power; however, even when women have some say in decisions, the final decision often ultimately rests with the man [22]. In these instances, efforts to improve women’s dietary diversity are unlikely to gain traction without first understanding and addressing unquestioned norms about food distribution and allocation that disadvantage women, including norms that women eat last, are restricted from nutritious food during pregnancy and while breastfeeding, and have limited access to foods with high prestige and nutritional value [[23], [24], [25]]. Without clear efforts to assess relevant norms and address them through innovative, community-led, proactive intervention strategies, consideration of social norms will continue to be relegated to post hoc discussion of the barriers or missed opportunities that have limited the effectiveness of nutrition interventions [19].

To move beyond the expectation that solely providing nutrition information to women, and even to other family and community members, will be sufficient to overcome the constraints that social norms place on women’s agency to improve nutrition, we need a greater understanding of the role of norms. This calls for practical methods and tools to identify the norms that impact nutrition and agency, and to assess whether intervention strategies are successful in influencing normative beliefs and agency as well as nutrition outcomes. A crucial initial step in understanding social norms related to nutrition practices is to conduct a “norms diagnosis” to identify what norms are at play, who enforces them, and what rewards and sanctions are associated with adherence. For instance, tools such as the Social Norms Exploration Tool (SNET [26]) and the Social Norms Analysis Plot (SNAP [27]) provide rapid, participatory, and practical approaches for collecting and analyzing information, aiding in the design of interventions, policies, and programs and adaptation to local contexts. It is equally vital to consult women about the social norms that enable or restrict their agency. With their input, effective intervention strategies can be developed and tested. Improved approaches to measuring social norms will make it possible to assess whether these interventions impact on nutrition behaviors and outcomes, and whether these impacts are mediated by shifts in agency and normative beliefs.

## Social Norms in Action: Snapshot from Niger

The United States Agency for International Development (USAID) Kulawa project, implemented by Save the Children and a consortium of organizations with funding from USAID/Niger, offers an example of the value of assessing social norms related to nutrition at the project design phase [28,29]. USAID Kulawa, part of the USAID Resilience in the Sahel Enhanced II Health Services Delivery activity in Niger, aims to improve access to and use of quality health services while strengthening ownership and management by communities, local government, and service providers. Kulawa means “to care for” in Hausa, and the project focuses on bridging the equity gap in maternal, newborn, and child health, family planning/reproductive health, and nutrition service access and utilization. To achieve these goals and build on local knowledge and capacity, USAID Kulawa prioritizes community participation and engagement in identifying and overcoming social and behavioral barriers preventing the adoption of better health practices. USAID Kulawa engages in a community-led development process using the proven Community Action Cycle [30] to increase demand for and use of quality health services and adoption of recommended health and nutrition behaviors at the community level. This approach, which has been used across West and Central Africa [31,32], empowers communities to identify and then analyze their health problems, prioritize action, develop community action plans, and monitor and evaluate progress over time.

In Niger, less than a quarter of children aged <6 mo are exclusively breastfed, and most are fed water and other liquids such as local teas and infusions in the first few months [33,34]. Moreover, exclusive breastfeeding is alarmingly low in the Maradi, Tillberi, and Zinder regions where USAID Kulawa works (36.2%, 8.8%, and 16.2%, respectively) [35]. The USAID Kulawa team applied the SNET to better understand whether and how social norms influence exclusive breastfeeding in program communities and respond as relevant. To equip staff with the skills to identify and investigate social norms, project staff were trained to use the SNET. Subsequently, they pretested and refined the data collection instruments based on community feedback and validation, interviewed 216 married and unmarried women aged 15–24 y who were first-time parents from a total of 6 villages, and conducted 6 group discussions in each village. The project staff also used participatory learning exercises to engage reference groups – groups of people with whom women compare themselves and enforce the behavior – and explore social norms that influence behaviors of interest. The formative assessment report, with detailed methodology and findings, has been published elsewhere [29].

First-time mothers most often identified their mothers-in-law and husbands as reference groups; some also included health workers. Social norms were among the most common drivers or influences of exclusive breastfeeding, including what a child should be fed, who decides what and when a child is fed, and women’s roles. These norms are deeply rooted and entrenched in the community’s traditions and customs. “Exclusive breastfeeding goes against our culture…There are decoctions and porridge that must be given to the child for his well-being so that he becomes strong. It is for the child to be in good health. They are milk supplements. (first-time mother age 15–24).” Many people explained that these norms are widespread, so others expect families to follow traditions. “Exclusive breastfeeding is not generally practiced in our society and this is since our ancestors…The community thinks that the baby will suffer a lot if it is fed only breastmilk. (first-time mother age 15–24).” It is also customary, according to religious tradition, to provide prophetic products (such as holy water, Zamzam, and dates) to the newborn infant.

Participants shared that the mother-in-law upholds these norms and determines what and when the child is fed. Although men have influence in the household, on this topic the husband follows his own mother’s decisions. “The mother-in-law exerts a strong influence on her son’s wife …as she is the husband’s mother and her decisions are unquestionable for fear of reprisals and threats of divorce. (reference group of first-time mother age 15–24).” “Husbands support their mothers’ decisions out of respect for social and religious norms. (reference group of first-time mother age 15–24).”

Furthermore, social expectations related to power and the division of labor limit what mothers can do, even if the family were to approve of exclusive breastfeeding. Heavy expectations for women to contribute to the family through both domestic and field work means “...mothers do not have the time to breastfeed the baby well and leave him at home with his grandmother. (first-time mother age 15–24).” Norms related to women’s power in the household are enforced by social sanctions that prevent a mother from going against the expectations of her mother-in-law, fearing insults and curses. A husband might divorce or mistreat a woman who is deemed insubordinate to her mother-in-law or husband. “The girl who practices exclusive breastfeeding will be treated as crazy, be seen badly by the neighbor, and the in-laws will reprimand her as if she does not treat her child well. (reference group of first-time mother age 15–24).”

These findings reveal how strongly social norms can influence women’s agency to breastfeed exclusively. Despite the important role of social norms in behavior change, programs in Niger, as in most countries, typically promote exclusive breastfeeding during antenatal care counseling with pregnant women or via group education sessions with pregnant women and mothers, ignoring the importance of other social actors. USAID Kulawa’s social norms exploration also suggests that it is crucial to consider norms related to gender roles that determine how women use their time.

## Fostering Nutrition Agency Through Social Norms-Aware Programming

Programs seeking to foster nutrition agency should delve into understanding the social context and norms that influence women’s agency, as well as support conditions that raise women’s awareness of their options, enable them to establish their own goals and facilitate their access to internal and external resources to achieve those goals, including the capacity to resist or manage pushback. In this section, we outline programmatic considerations and guidelines for “norms aware” programming. We also describe and illustrate, with examples from USAID Kulawa and other projects, several principles of good quality social norms programming that could support effective and culturally appropriate efforts to design norms-responsive nutrition programming.

Norms shape women’s choices and actions by influencing the options they perceive as available and desirable, by determining the resources they can draw on to achieve their choices, by the positive or negative sanctions women experience when they follow or violate social expectations. With a contextual understanding of the social norms related to specific nutrition behaviors, such as the insights from the USAID Kulawa social norms exploration, program staff can work with communities to address these norms. It is often easier to shift norms that are already beginning to change, which can be identified in consultation with communities, using social norms exploration tools such as the SNET [26] and SNAP framework [27] or ongoing program monitoring guidance [36]. Thus, a deep understanding of norms can help programs focus on “cracks” in norms, where individuals are willing to speak positively about their experiences experimenting with new behaviors, and identify positive deviants in the community who can serve as role models in promoting nutrition behaviors and positive norms. Social norms interventions typically work across levels of the socio-ecologic system to engage individuals, couples, households, communities, leaders, health providers, and the broader system through interpersonal communication, reflective dialog, or mass media campaigns. As seen in the USAID Kulawa example, it is important not only to support new mothers, but also to engage their mothers-in-law, husbands, and the broader community who may criticize or support new child-feeding practices. For example, based on the formative research findings, USAID Kulawa is working with imams to organize sermons on inequalities related to norms, behaviors, and practices that hinder access to services or fuel discrimination and deprivation. Imams leverage verses from the Koran that support family health and well-being to encourage the adoption of priority nutrition behaviors such as exclusive breastfeeding. By working across the socio-ecologic system, programs can be positioned to confront the power imbalances related to gender and social hierarchy that exist and interfere with child-feeding practices and other nutrition behaviors. In addition, effective social norms-shifting programs can promote ongoing**,** critical reflection that goes beyond training, one-off campaigns, or ad hoc outreach, often in small group settings [37].

A “norms aware” program in Niger could involve engaging both husbands and mothers-in-law, recognizing their influence on both their daughters-in-law and sons [9,20,38]. The problems first-time mothers experience with breastfeeding and lack of knowledge can be addressed during antenatal care counseling and mentorship. However, addressing the norms related to child feeding enforced by mothers-in-law and husbands through sanctions will require a broader lens. USAID Kulawa’s findings suggest that the program should consider factors that affect mothers beyond norms – such as experiencing pain when breastfeeding – which limits their ability to breastfeed, consider the influence of mothers-in-laws and husbands, and prepare women to navigate sanctions for violating infant feeding norms. Failing to address norms and encouraging mothers to act on their agency exposes mothers to sanctions and burdens them unfairly – forcing them to navigate conflicting guidance from health workers and their families. Moreover, it leaves them without the skills or support they need to navigate social pressures that may interfere with achieving their goals. This approach does a disservice to women and is likely to be particularly ineffective for those with fewer resources and social assets to call upon. Understanding the specific norms and influencers at play can shape more effective programs to tackle the power of social norms to shift behavior. For this reason, USAID Kulawa, in collaboration with community leaders, facilitates nutrition dialog sessions for young mothers and their mothers-in-law on maternal nutrition, exclusive breastfeeding, infant and young child nutrition, consumption of micronutrient-rich foods, growth monitoring, and hygiene. These dialog sessions, led by community health workers, equip mothers-in-law with the skills necessary to support young breastfeeding mothers in their households.

Social norms shift when it becomes apparent that old norms are no longer relevant or are harmful, or when enough people see others who matter to them changing their behavior. Thus, nutrition programs can consider strategies that facilitate modeling and reflection of desired behaviors, public testimony about the value of a new norm and behavior, and activities to diffuse these new ideas beyond direct program recipients. Programs addressing social norms differ from those targeting knowledge and attitudes because they seek change at the community level rather than (or in addition to) the individual level. In the realm of nutrition programming, this might mean including family members in some of the home visits to mothers or community education sessions, or creating tailored media content for family members and their influencers. Alive and Thrive, a large-scale multichannel intervention that addressed social norms through these approaches, demonstrated positive impacts on norms and nutrition outcomes [39]. Other promising strategies involve discussion groups for grandmothers, engaging men in reflection to surface and address norms, community dramas, songs, and games, and amplifying the voices of those individuals who set and enforce social norms [38]. In USAID Kulawa, community-level nutrition activities are reinforced by their counterparts from a complementary intervention known as Husband Schools that involves men in increasing the demand and use of reproductive, maternal, neonatal, and child health and nutrition services at the household and community levels [40]. With the support of local health workers, Husband Schools offers opportunities for men to create a healthier family ecosystem and mobilize their peers to adopt recommended practices. Members engaged in these Husband Schools learn about various health topics, play roles as community promoters and service linkers, and propose individual and community activities to increase the use of health services and good practices.

There is a growing body of evidence on what to avoid when integrating a norms perspective into behavior change initiatives that nutrition programs can take to heart. First, programs should not be top–down [11], rather they should be designed and implemented in collaboration with communities [[41], [42], [43]]. Nutrition programs have a long history of effective community engagement [37,[44], [45], [46]] and are thus well-positioned to learn about and consider social norms relevant to local contexts. Second, although it is common to focus on the negative consequences of a behavior, this can unintentionally reinforce that behavior by making it seem widespread. Instead, finding and amplifying protective norms that build on existing positive values can be the simplest approach to desired behavior change [11,47]. For instance, in the case of complementary feeding, the emphasis should be placed on parents undertaking the necessary actions to provide their child with the best start in life. In Niger, USAID Kulawa reinforced protective norms by using social behavior change tools that leverage religious texts and speak to the importance of family and harmony. Finally, it is important to remember that social norms may influence nutrition behaviors directly or indirectly [20,38,48]. Social norms related to women’s productive role in the family, for example, may influence breastfeeding indirectly by limiting the time she has available to breastfeed, as was the case in Niger. Expectations about what children should be fed, on the other hand, may influence infant feeding directly, with clear sanctions, such as emotional or physical abuse or blaming mothers if the child falls ill.

Addressing social norms – whether uplifting positive ones or shifting those that are harmful – takes time and there is no set recipe for success. Programs and research should engage community members actively throughout the process. Research teams can engage communities in research design, data collection, analysis, and interpretation. Similarly, effective interventions typically involve communities in program design, implementation, monitoring, and evaluation, drawing on existing resources such as the Community Action Cycle [30]. Moreover, it is imperative that research or program strategies do not inflame harmful norms or put women in danger. Applying a lens of collaborating, learning, and adapting will help programs anticipate, plan for, monitor, and mitigate pushback and any unanticipated harmful effects of their work to shift norms. It is particularly important to monitor the skills and attitudes of facilitators, as they are critical catalysts in the norms-shifting process. Qualitative approaches, such as reflection meetings, observation, and interviews with implementers, are well suited to identify signs of whether perceptions of the norms relevant to program outcomes are weakening, strengthening, or not changing at all, and whether new norms are emerging [36,49]. This information can generate important insights for program action. Before implementation, it is also important to validate activities addressing norms with the target audience to ensure that these activities are culturally appropriate. Additionally, documenting the process and outcomes of social norms programs is crucial for building a body of evidence [50] and, eventually, best practices to address the social norms and foster nutrition agencies. An emerging consensus suggests that norms can be measured both directly and indirectly using different formats, including single-item questions, scales, vignettes, focus group discussions, in-depth interviews, and participatory methods [51]. This investment of time and resources in documentation can yield long-term benefits beyond individual programs.

In conclusion, it is time to recognize, acknowledge, and seek to support communities in addressing the normative constraints and barriers that women face on their journey to better nutrition. Actively engaging the social context that influences women’s nutrition agencies holds the promise of achieving long-lasting, widespread behavior change, a goal that has often proven elusive when programs focus singularly on the knowledge and attitudes of individual women. Integrating a “norms aware” approach into research and programs, with small but significant adjustments to program models as offered in the guidelines above, holds the promise of better adaptation to local contexts and lasting impacts on nutrition and health, as well as agency and other aspects of well-being. Furthermore, integrating norms perspectives into research and programs is more achievable now, given the array of resources available for identifying social norms and reference groups that enforce them, designing norms-aware programs, and monitoring and evaluating norms-shifting programs.

## Author contributions

The authors’ responsibilities were as follows – FEW, KLD, LS, RL: conceptualized the commentary; all authors (FEW, KLD, LS, RL, MP, MD, AB): contributed to the writing and revision of the manuscript; FEW: had primary responsibility for the final content and led the submission process; and all authors: read and approved the final manuscript.

## Conflict of interest

The authors report no conflicts of interest.
